# The Reduction in the Mitochondrial Membrane Potential in Aging: The Role of the Mitochondrial Permeability Transition Pore

**DOI:** 10.3390/ijms241512295

**Published:** 2023-08-01

**Authors:** Hagai Rottenberg

**Affiliations:** New Hope Biomedical R&D, 23 W. Bridge Street, New Hope, PA 18938, USA; rotteh@hotmail.com

**Keywords:** aging, mitochondria, membrane potential, permeability transition pore, *C. elegans*

## Abstract

It is widely reported that the mitochondrial membrane potential, ∆Ψm, is reduced in aging animals. It was recently suggested that the lower ∆Ψm in aged animals modulates mitochondrial bioenergetics and that this effect is a major cause of aging since artificially increased ∆Ψm in *C. elegans* increased lifespan. Here, I critically review studies that reported reduction in ∆Ψm in aged animals, including worms, and conclude that many of these observations are best interpreted as evidence that the fraction of depolarized mitochondria is increased in aged cells because of the enhanced activation of the mitochondrial permeability transition pore, mPTP. Activation of the voltage-gated mPTP depolarizes the mitochondria, inhibits oxidative phosphorylation, releases large amounts of calcium and mROS, and depletes cellular NAD^+^, thus accelerating degenerative diseases and aging. Since the inhibition of mPTP was shown to restore ∆Ψm and to retard aging, the reported lifespan extension by artificially generated ∆Ψm in *C. elegans* is best explained by inhibition of the voltage-gated mPTP. Similarly, the reported activation of the mitochondrial unfolded protein response by reduction in ∆Ψm and the reported preservation of ∆Ψm in dietary restriction treatment in *C. elegans* are best explained as resulting from activation or inhibition of the voltage-gated mPTP, respectively.

## 1. Introduction

**Figure 1:**
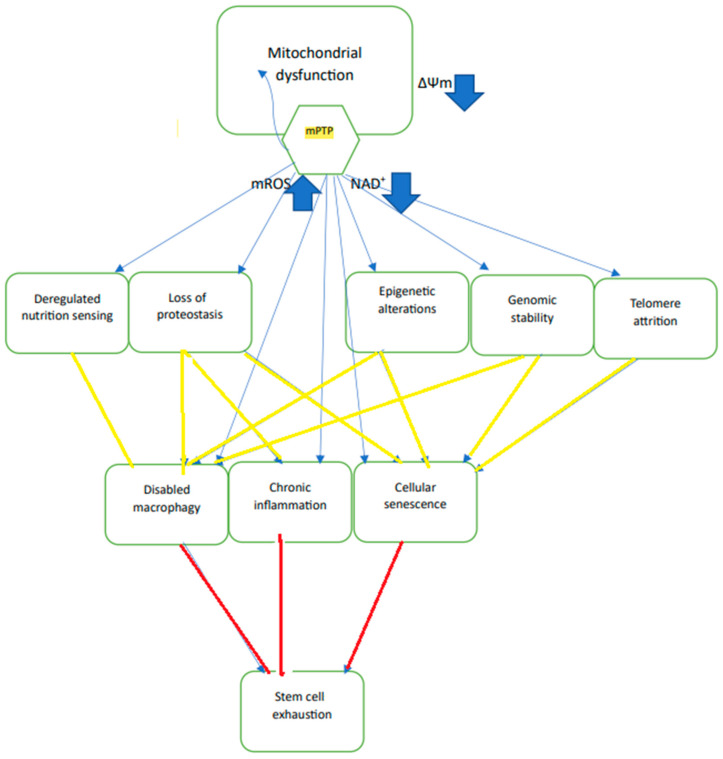
The activation of mPTP depolarizes the mitochondrion and directly negatively affects most “hallmarks of aging” largely by releasing large amounts of mROS and depleting cellular NAD^+^ (blue arrows). These processes, in turn, negatively affect other “hallmarks of aging” (yellow arrows) that would eventually lead to stem cell exhaustion (red arrow).

Aging can be defined as a time-dependent functional decline that is strongly associated with cellular damage in critical organs. In animals, these time-dependent processes can manifest in several phenomena, distributed in several tissues and organs, that have been summarized as “hallmarks of aging” [1,2]. Hallmarks of aging have been defined as phenomena that are observed in aged animals, with their enhancement leading to accelerated aging, while their inhibition retards the aging process. It is well-documented that these are not independent of each other but are connected in a complex network of interactions that are not fully resolved. Among the twelve identified hallmarks of aging by Lopez-Ortin et al. [1,2], “mitochondrial dysfunction” is, arguably, the most strongly connected with other hallmarks of aging. The most frequently observed manifestation of mitochondrial dysfunction in aging is the excess production of mitochondrial reactive oxygen species, mROS [3], which negatively affects most of the other hallmarks of aging [4] i.e., genomic instability [5], telomere attrition [6], loss of proteostasis [7], deregulated nutrient sensing [8], cellular senescence [9], inflammation [10], stem cell exhaustion [11], and loss of epigenetic information [12] (Figure 1). It was suggested early on, therefore, that excess mROS is the main driving force for aging, as postulated by the mitochondrial free radical theory of aging [13,14]. We have suggested previously that the principal pathway for the generation and delivery of excess mROS in aging cells is the opening of the mitochondrial permeability transition pore (mPTP) [15,16]. Since mPTP activation was shown to accelerate aging, and mPTP inhibition was shown to retard aging, enhanced activation of mPTP meets the definition of a “hallmark of aging”.

The mitochondrial permeability transition pore (mPTP) is a protein complex located in the mitochondrial inner membrane. When activated, mPTP forms a mega-channel that is non-selective and is permeable to ions and solutes up to 1500 kDA in size [17,18]. However, the channel can exhibit a range of conducting states from about 45 pS up to >1000 pS. The channel is voltage-gated, activated by lowering ∆Ψm and inhibited by raising ∆Ψm, and is also inhibited at low matrix pH. While there are many agents that can activate the channel, physiologically it is mostly activated by mitochondrial matrix calcium overloading. Another physiologically activating agent is the excess production of mitochondrial reactive oxygen species (ROS) that leads to oxidative stress in the mitochondrial matrix. Oxidative stress results in oxidation of pyridine nucleotides that activate mPTP. Oxidative stress in the mitochondrial matrix also results in the oxidation of a number of SH residues on the channel proteins, as well as of additional proteins that are associated with the channel, and this oxidation of SH residues also activates mPTP. Recent evidence suggests that the main components of the channel are ATP synthase (F1F0) and adenine nucleotide translocase (ANT). Additional proteins that interact with the channel’s proteins control their activity. The most important mPTP-associated protein is cyclophilin D (CypD) that binds to and activates mPTP. In addition, the outer membrane protein VDAC can also activate mPTP, most likely by interacting with ANT at contact sites between the inner and outer membranes. While the exact mechanism of conductance through the channel has not yet been elucidated, it is apparent that the conductance pathways through ATP synthase, ANT or their interface allow for the formation of variety of channels with different conductance and variable open durations. Post-translational modifications and binding of ions to the proteins that control mPTP activity, and particularly to CypD, that control the channel activities of both ATP synthase and ANT, contribute to the many pathways that allow for the activation or inhibition of the channel. Thus, phosphorylation at one site of CypD can inhibit the channel and phosphorylation at another site can activate the channel. Acetylation of CypD activates the channel and deacetylation by sirt3 inhibits the channel. Channel activity can also be controlled by small molecules that bind to CypD and inhibit channel opening, such as cyclosporin A. In contrast, free fatty acids, most likely through their interaction with ANT, activate the channel. Other inhibitors of ANT either activate (atractyloside) or inhibit the channel (bonkrekic acid), while the ANT substrate ADP also inhibits the channel. The activation of mPTP by excess mROS can be inhibited by strong antioxidants, such as mitoQ and quercetin.

Partial opening of mPTP appears to be beneficial since the release of mROS and Ca serve as signals to the nucleus to activate protective mechanisms, such as the mitochondrial unfolded protein response (UPRmt), that activate the synthesis of chaperones, such as HSP60, HSP75, HSP90 (TRAP1), that are transported to the mitochondria where they inhibit mPTP. Other protective mechanisms are also activated, such as NRF-2 that increases the expression of enzymes that protect against oxidative damage, and PGC-1α that increases mitochondrial biogenesis. Because partial opening does not allow the release of respiratory substrates, ∆Ψm is quickly restored after the release of the activating excess ROS and calcium and the channel closes. Full opening of the mPTP releases most matrix metabolites, including respiratory substrates, mROS, calcium, NAD^+^, and glutathione, while allowing protons to flow into the mitochondria. As a result, ∆Ψm collapses and ∆pH vanishes, and the collapse of the protonmotive force inhibits oxidative phosphorylation. On prolonged opening, solutes from the cytoplasm flow into the mitochondria resulting in large-scale swelling of the inner membrane causing the outer membrane to rupture, releasing intermembrane space proteins, like cytochrome c, that induce apoptosis. Moreover, the release to the cytosol of excess ROS, calcium, and NAD^+^ the hydrolysis of the released NAD^+^ by CD38 [19], disrupt cellular homeostasis and increase oxidative stress that damage cell proteins, nuclear DNA, ion channels, transporters, and membrane phospholipids, all of which accelerate aging. Although the release of excess matrix calcium and increase in the matrix pH would favor channel closure, the inability to increase ∆Ψm because of the escape of respiratory substrates that normally pump protons and generate ∆Ψm would prevent channel closure. The damage caused by prolonged mPTP opening is normally mitigated by the process of mitophagy that removes damaged mitochondria and is activated by mPTP-induced collapse of ∆Ψm and the release of ROS. Thus, the limited extent of mitophagy is beneficial and retards aging and slows aging-driven degenerative disease. However, extensive mitophagy would deplete the cell mitochondria. Therefore, prolonged pore opening in a large number of mitochondria in the cell can lead to cell death by necrosis, apoptosis, or similar pathways. In aging, many of the protective mechanisms described above are inhibited, including mitophagy, UPRmt and sirt3 activity; the potential threshold for channel opening is reduced, and calcium homeostasis is compromised. These effects of aging, together with NAD^+^ depletion and excess production of mROS, enhance the activity of mPTP, thus accelerating aging and age-driven degenerative disease since both excess ROS and NAD^+^ depletion affect many of the hallmarks of aging directly and nearly all of them indirectly [15,16,20] (Figure 1).

Another often reported mitochondrial dysfunction in aging is reduction in the magnitude of ∆Ψm (reviewed in [21,22,23]. It was recently suggested that the reduction in ∆Ψm in aged animals is a major cause of aging [23,24,25,26,27]—this claim was supported by the demonstration that the optogenetic-induced increase of ∆Ψm increased *C. elegans* lifespan [25]—and that calorie restriction that increases *C. elegans* lifespan restores ∆Ψm in aged animals [26]. In animal cells ∆Ψm varies within a narrow range (−130 to −180 mV); many metabolic functions affect the magnitude of ∆Ψm. The ATP synthase that enables the synthesis of ATP is driven by the protonmotive force, which is composed of ∆Ψm and ∆pH. The flow of protons from the intermembrane space to the matrix during oxidative phosphorylation thus reduces ∆Ψm. The transition from a minimal rate of ATP synthesis (“state 4”) to a maximal rate of ATP synthesis (“state 3”) is associated with a 25–35 mV reduction in ∆Ψm. In addition, ∆Ψm depends on the electron transport substrate that drives the generation of ∆Ψm, the activity of electrogenic or proton-coupled transporters, and the activity of uncoupling proteins, all of which may reduce the magnitude of ∆Ψm [28]. The upper limit of ∆Ψm is determined by the magnitude of the proton leak through the mitochondrial inner membrane. Since the proton leak increases exponentially with ∆Ψm [29], it is not possible to significantly hyperpolarize the mitochondrial inner membrane. There is also a lower limit to the magnitude of ∆Ψm in viable mitochondria. The intermediate metabolism in the mitochondrial matrix depends on the electrogenic and proton-coupled transporters, and mROS signaling depends on ∆Ψm [30]. In circumstances where the electron transport system cannot generate a protonmotive force large enough to drive ATP synthesis, ATP generated by glycolysis can reverse ATP synthase, and ATP hydrolysis by ATP synthase can increase ∆Ψm to a level that is sufficient to maintain functional mitochondria [31]. If cellular ATP concentration is not sufficient to restore the physiological level of ∆Ψm, the process of mitophagy will eliminate depolarized mitochondria [16], and, if a large number of mitochondria in the cell become depolarized, cell death by necrosis or apoptosis will follow [32].

In this review, I examine, critically, and in detail, the reported reduction in ∆Ψm in aged animals and conclude that the most reasonable explanation of most of these observations is the widely reported enhancement of the activation of mPTP in aging animals (reviewed in [15,16,33,34,35,36,37]. In addition, I discuss the evidence that the lifespan extension of *C. elegans* obtained by the optogenetic increase of ∆Ψm [25] most likely resulted from the predicted inhibition of the voltage-gated mPTP, and that the restoration of ∆Ψm by calorie restriction in *C. elegans* [26] also resulted from inhibition of mPTP.

## 2. The Effect of Aging on the Magnitude of the Mitochondrial Membrane Potential in Animals

It is widely reported that the magnitude of ∆Ψm in old animals is lower than that of ∆Ψm in young animals, as reviewed in [21,22,23]. Table 1 summarizes representative studies of the effect of aging on ∆Ψm in different animals, different cell types, and using different methods. To interpret these studies correctly, it is necessary to examine the protocols of ∆Ψm measurements in these studies.

The determination of ∆Ψm in isolated mitochondrial is relatively simple. A permeable cation is allowed to equilibrate across the mitochondrial inner membrane and reach electrochemical equilibrium; the concentrations of the cation in the mitochondrial matrix and the medium at equilibrium is determined, and ∆Ψm is calculated from the concentration ratio according to the Nernst equation: ∆Ψm = −60 × log([C] in/[C] out) [38]. In the most recent studies of ∆Ψm, lipophilic membrane-permeable cations are employed. In one method, the distribution of radioactively labeled lipophilic cations, such as TPP^+^, between the mitochondria and the medium is determined after quick separation of the mitochondria and the medium (e.g., by filtration or centrifugation), and ∆Ψm is calculated from the ratio C_pelet_/C_medium_. Another method used is the measurement of the mitochondrial uptake of TPP^+^ from the medium, as measured by a TPP^+^ electrode immersed in the mitochondrial suspension and calculation of ∆Ψm from the amount of TPP^+^ taken by a known volume of mitochondria. More frequently, the uptake of lipophilic cationic fluorescent dyes is used to estimate the relative magnitude of ∆Ψm. Measuring ∆Ψm in isolated mitochondrial suspension with a fluorescent dye in most routine measurements depends on the fact that, at high matrix concentration, the dye aggregates on the inner membrane surface and the fluorescence is quenched; the amount of accumulated dye is estimated from the residual fluorescence of the free dye that remains in the suspending medium. However, the aggregated dye is not free in the matrix, and, therefore, an exact calculation of the magnitude ∆Ψm from the extent of quenching must be corrected for the aggregation or calibrated separately.

Most of the reported measurements of ∆Ψm in old animals were measured in intact cell suspensions where ∆Ψm was estimated from the fluorescence of lipophilic ∆Ψm indicators that were accumulated by the cells (e.g., R-123, DioC_3_(6), TMRE). This method is not as simple as the measurement of ∆Ψm in isolated mitochondria. In this method, it is necessary to use very low dye concentrations to prevent aggregation and quenching in the mitochondrial matrix since this would saturate the fluorescence and the cell fluorescence would no longer be proportional to ∆Ψm. Other factors also need to be considered, e.g., the mitochondrial content of the cells (which may be reduced in aging), the mitochondrial matrix volume, and the plasma membrane potential that drives the accumulation of the dye into the cell [39,40,41]. The fluorescence maximum of the lipophilic cation JC-1 shifts from green fluorescence of the monomer to red fluorescence of the aggregated dye, and, therefore, ∆Ψm is estimated from the ratio of red/green fluorescence [42]. However, a carefully selected concentration is required to ensure that the transition occurs over a range of concentration that is sensitive to high ∆Ψm. In this case too, if the concentration in the medium is too high, all the mitochondria with significant ∆Ψm will have red fluorescence, regardless of the value of ∆Ψm, and only depolarized mitochondria will have green fluorescence.

Unfortunately, many of the studies of ∆Ψm in cells from old animals did not use the optimal dye concentrations and did not take into consideration all the factors that can influence the fluorescence, which renders their interpretation more difficult. Most importantly, all the available studies, either from cells or isolated mitochondria, reported the average ∆Ψm, but not the values from single mitochondria, so it is quite possible that the lower average values of ∆Ψm reported for cells or isolated mitochondria from old animals represent a mixture of depolarized mitochondria and mitochondria with high ∆Ψm [43]. As discussed below, there is not a single study that shows that there is a normal distribution of ∆Ψm values in mitochondria from aged cells, or that the lower ∆Ψm is not a mixture of fully polarized and depolarized mitochondria.

Kokoszka et al. [44] measured ∆Ψm in isolated liver mitochondria from young and old mice. Using a TPP^+^ electrode, they observed a small difference between mitochondria from young mice (−180 mV) and old mice (−170 mV). Kokoszka et al. [44] also reported the inhibition of electron transport and increased oxidative stress. Most importantly mitochondria from old mice also exhibited enhanced activation of mPTP. It should be clear that, since the measured ∆Ψm is an average of a large number of mitochondria, it is possible that ∆Ψm is not uniformly distributed. If that was the case, a reduction in the average ∆Ψm from −180 mV to −170 mV in mitochondria from old mice may indicate that 32% of the mitochondria are depolarized (∆Ψm = 0), and that 68% maintain the same ∆Ψm as young mice (−180 mV). This estimate was obtained by the following calculation based on the Nernst equation (see above): since 180/60 = 3, a potential of −180 mV corresponds to a ratio of TPP^+^_in_/TPP^+^_out_ of 1000 (log3), while the ratio for −170 mV would be 676 (log2.83). We assume that this latter ratio is a weighted average of the two fractions of mitochondria: high potential, −180 mV (H), and low potential, 0 mV (L). Therefore, 1000 H + 1 L = 676, while H + L =1. Solving these equations for L yields L = 0.324, and, therefore, H = 0.676. This estimate is not very sensitive to the absolute values of ∆Ψm. If the potential for mitochondria from young mice was −170 mV and that for old mice −160 mV, the fraction of depolarized mitochondria would be 31%. Therefore, it is possible to explain the small reduction in ∆Ψm in liver mitochondria from old mice as resulting from the aging-induced enhanced activation of mPTP that resulted in a larger fraction of depolarized mitochondria. In the liver (or other organs), mitochondria with fully open mPTP will be cleared by mitophagy [16], so this fraction should normally be small. However, aging inhibits mitophagy; so, in the aged liver, this fraction could be larger. Moreover, in isolated mitochondria where mitophagy is absent, the fraction of depolarized mitochondria increases with time and only carefully selected isolation and incubation protocols retard this process.

The enhanced activation of mPTP in isolated liver (and brain) mitochondria from old mice was confirmed independently by the measurement of the threshold for calcium-induced calcium release [45]. This common assay of mPTP activation does not really distinguish between mitochondria that are more sensitive to calcium-induced activation of mPTP (the standard interpretation) and a mitochondrial preparation that already includes depolarized mitochondria that cannot, therefore, accumulate calcium.

LaFranca et al. [46] measured ∆Ψm in isolated mitochondria from rat cortical and striatal neurons from young and old rats. Similar to Kokuszka et al. [44], they also used a TPP^+^ electrode and reported a relatively small reduction in the average ∆Ψm, 10–15 mV, in neurons from old rats. Similarly, they also reported enhanced activation of the mPTP in cortical neurons from old rats. Very recently, Gainutdinov et al. [47] also reported that brain mitochondria from old rats exhibited lower ∆Ψm as measured by the quenching of safranine fluorescence, and that brain mitochondria from old rats also exhibited enhanced activation of mPTP.

Thus, experiments with isolated mitochondria, either from mouse liver or rat brain, suggest small reductions in the average ∆Ψm in mitochondria from old animals (10–15 mV). Since this small reduction in ∆Ψm was, in all cases, associated with enhancement of the activation of mPTP, it is possible that one explanation of the observed small reduction in the magnitude of ∆Ψm resulted from depolarization of a fraction of the mitochondria by mPTP opening, but only determination of ∆Ψm in individual mitochondria can test this explanation.

The earliest reports that there was a reduction in ∆Ψm when measured in intact hepatocytes from old rats, were from the Bruce Ames laboratory. In their earliest paper, Haggen et al. [48] used the accumulation of the radiolabeled cation TPP^+^ by hepatocytes to calculate ∆Ψm. They reported that, in contrast to hepatocytes from young mice that have a uniformly high ∆Ψm (−154 mV), hepatocytes from old mice had much lower average ∆Ψm (101 mV) and could be separated into three fractions: a small fraction of cells with high ∆Ψm (same as young, −154 mV), a small fraction with intermediate ∆Ψm (−93 mV), and a large fraction with low ∆Ψm (−70 mV). It is quite possible that, even in the cell fraction with the low averaged ∆Ψm, there was a mixture of mitochondria with high ∆Ψm and depolarized mitochondria. It is also possible that the density of mitochondria in hepatocytes from old rats is lower than that of hepatocytes from young rats. In most of their other experiments Haggen et al. [48] used the fluorescent potential indicator rhodamine-123 and measured hepatocyte fluorescence by flow cytometry, a method that was also used in subsequent studies [49,50]. They reported >50% reduction in the fluorescence intensity in hepatocytes from old mice compared to hepatocytes from young mice. The dye concentration used in these studies was very high (26 μM), orders of magnitude higher than the recommended dye concentrations needed to avoid aggregation and quenching. It was previously demonstrated that only when the external concentration of the dye is in the low nM range is the fluorescence of the dye in the mitochondrial matrix not quenched, and the magnitude of ∆Ψm is proportional to the fluorescence intensity [39,40,41]. Therefore, it is quite clear that, in the experiments of Haggen et al. [48,49,50], the fluorescence intensity is saturated and, therefore, insensitive to the magnitude of ∆Ψm. However, since the dye will only accumulate in mitochondria that have a ∆Ψm >> 0, the fluorescence intensity is proportional to the number of mitochondria in the cells that are not depolarized and retain high ∆Ψm. If aging increases the number of mitochondria with activated mPTP, which are, therefore, depolarized, and decreases mitochondrial density due to clearance of depolarized mitochondria by mitophagy, the fluorescence intensity will be lower in aged hepatocytes. Haggen et al. [48,49,50] did not measure the mitochondrial density in hepatocytes from old and young rats. Therefore, these measurements suggest that, in isolated hepatocytes from old mice, ~50% of the mitochondria were either depolarized or were further cleared by mitophagy. Haggen et al. [48,49,50] reported other experiments with aged hepatocytes that support this interpretation. They showed that the reduction in ∆Ψm was associated with increased production of mROS, which is known to be associated with enhanced activation of mPTP. Many of the studies of mitochondrial dysfunction in aging (and degenerative diseases) report an association of increased mROS production with reduced ∆Ψm [51]. Since it is well established that, in general, mROS production increases exponentially with ∆Ψm [52,53,54], the unusual association observed in aging animals of high mROS and low ∆Ψm most likely results from the enhanced activation of mPTP.

Cavazzoni et al. [55] measured ∆Ψm in hepatocytes from young and old rats with R-123 by flow cytometry, at relatively low concentration of R-123 (130 nM), which they claim was not saturating under their conditions. In contrast to Haggen et al. [48,49,50], they did not observe a significant difference in ∆Ψm between hepatocytes from young and old rats. However, the standard deviations in their measurements were very high ± 45 mV, possibly obscuring a small difference between young and old rats.

Rottenberg and Wu [56] estimated the relative magnitude of ∆Ψm in intact lymphocytes from young and old mice. They used very low concentrations of the lipophilic dye DiOC_3_(6), well below the saturating concentration [39], and measured fluorescence by flow cytometry. They found that ∆Ψm was significantly lower in lymphocytes from old mice and that the fraction of cells with depolarized mitochondria was much larger in lymphocytes of old mice. In addition, the rate of respiration was inhibited in lymphocytes from old mice. Most significantly, cyclosporin A, an inhibitor of mPTP, restored both the rate of respiration and ∆Ψm in lymphocytes from old mice, thus proving that the lower ∆Ψm in lymphocytes from old mice was the result of enhanced activation of mPTP. Interestingly, the activation of mPTP in lymphocytes from old mice was largely confined to the older, memory T-cells [57], probably because most other types of lymphocytes have a relatively short lifespan.

Sugrue et al. [58] measured ∆Ψm in senescent and non-senescent PC12 cells using confocal microscopy and the fluorescent probe CMTMR and reported that the fluorescence was lower in senescent cells. The measurement of ∆Ψm by CMTMR is controversial [22] because, unlike the measurement of ∆Ψm with other cationic fluorescent probes that is based upon equilibrium distribution of the free probe across the membrane, the cationic CMTMR, while accumulating in the matrix due to ∆Ψm, is covalently bound in the matrix. Therefore, the amount of bound probe is not proportional to the magnitude of ∆Ψm; rather, it would be proportional to the number of mitochondria that have significant ∆Ψm because the probe would not accumulate in mitochondria where ∆Ψm = 0. Therefore, the results of Surge et al. [58] should be interpreted that, compared to non-senescent PC12 cells, the number of mitochondria with ∆Ψm >> 0 is smaller in senescent PC12 cells. Most importantly, Sugrue et al. [58] also showed that the effect of aging on CMTMR fluorescence was the result of the enhanced activation of mPTP in senescent cells since cyclosporin A restored CMTMR fluorescence to the same magnitude of non-senescent PC12 cells.

More recently, Durak and Turan [59] reported that in cardiomyocytes from aged rats, ∆Ψm was significantly lower than that of ∆Ψm in cardiomyocytes from young rats. In their measurements, they used the fluorescent probe JC-1 at a very high, clearly saturating concentration (4 μM). Therefore, their data must also be interpreted as resulting from an increased number of depolarized mitochondria in aged cardiomyocytes. Since they also reported increased production of mROS in aged cardiomyocytes, and that Liraglutide, a GLP-1R agonist, inhibited mROS production and restored ∆Ψm, their data are consistent with the interpretation that the increased depolarization of mitochondria in myocytes from old rats is the result of enhanced activation of mPTP.

Morris et al. [60] investigated the mitochondrial metabolism in old and young intestinal stem cells in *Drosophila*. They found that old intestinal stem cells exhibited a distinct Warburg-like metabolic shift, in which oxidative phosphorylation was inhibited and glycolysis was enhanced. This was largely the result of the inhibition of calcium uptake by MCU, which led to low activity of the ETC, resulting in inhibition of ATP synthesis. They reported lower membrane potential in old stem cells as measured by the fluorescence of TMRE (20 nM) from the mitochondria (labeled by mitoGFP). The average fluorescence intensity was ~20% lower in cells from old bees compared to young. While it is quite possible that such drastic metabolic shift was associated with lower ∆Ψm, the data shown report average cell fluorescence and it is not clear if the probe was equally distributed between the mitochondria in the cell. Morris et al. [60] also reported higher hydrogen peroxide production in old stem cells, so it is likely that mPTP activity was also enhanced in these cells.

Mansell et al. [61] studied hematopoietic stem cells (HSC) from young and old mice. They measured ∆Ψm by flow cytometry using TMRM (100 nM + Verapamil, to inhibit probe efflux) and reported lower ∆Ψm in HSC from old mice. However, the HSC population form of old mice exhibited two distinct fractions: a large fraction (~85%) with low ∆Ψm and a smaller fraction (~15%) with high ∆Ψm, like that of young cells. When separated using a cell sorter, the fraction with high ∆Ψm exhibited similar properties to young HSC, whereas the HSC with low ∆Ψm exhibited the distinct profile of old HSC. Moreover, when HSC from old mice were treated with MitoQ, a strong mitochondria-specific antioxidant, the fraction of high ∆Ψm was greatly increased, largely reversing the effects of aging on HSC. Since MitoQ was shown to inhibit mPTP [62], these finding are compatible with the interpretation that the reduction in ∆Ψm in HSC from old mice resulted from the enhanced activation of mPTP. Similarly, the antioxidant quercetin, which is also known to inhibit mPTP [63], restored ∆Ψm in aged porcine oocytes [64]

Perhaps the best evidence that the reduction in ∆Ψm in aged animals is the result of mPTP activation can be seen in the study of Zhang et al. [65]. They used JC-1 fluorescence to measure relative ∆Ψm in rat cardiomyocytes and reported a small reduction in ∆Ψm in cardiomyocytes from aged rats. They also reported increased mROS production, increased activation of mPTP, as well as enhanced frequency of mitoflashes (that indicate short opening of mPTP) in aged cardiomyocytes. They also showed that the tetra-peptide SS-31, which strongly binds to both ANT and ATP synthase (the major components of the mPTP complex [18]), inhibits many of the effects of aging on cardiomyocytes. In aged cardiomyocytes, SS-31 restored ∆Ψm, inhibited excess mROS production, inhibited mPTP activation, and inhibited excess activation of mitoflashes, providing strong evidence that the reduction in ∆Ψm in aged cardiomyocytes was the result of the enhanced activation of mPTP.

**Table 1:** Studies that reported reduced mitochondrial membrane potential, ∆Ψm, in aged animals. The table shows the species, tissue, method of measurement, and any reported treatment that restored ∆Ψm, or reported activation of mPTP. In those cases where ∆Ψm was calculated, the values of the measured difference are in mV. For the fluorescence ∆Ψm indicators, the % difference in fluorescence intensity or ratio is indicated. “Q” labeling indicates that the fluorescence measurement was in the quench mode.

System	Method	Probe	∆Ψm Difference Young–Old	Restoration of ∆Ψm	mPTP Activation	Reference
Mouse liver mitochondria	Cation distribution	TPP^+^ electrode	10 mV	Not tested	Yes	Kokoszka et al. [44]
Rat cortical neuron mitochondria	Cation distribution	TPP^+^ electrode	10–15 mV	Not tested	Yes	LaFranca et al. [46]
Rat brain mitochondria	Fluorescence	Safranin 2 μM (Q)	10%	Not tested	Yes	Gainutdinov et al. [47]
Rat hepatocytes	Cation distribution	TPP^+^ cell/medium distribution	84 mV	Not tested	Not tested	Haggen et al. [48]
Rat hepatocytes	Fluorescence	R-123 26 μM (Q)	>50%	Not tested	Not tested	Haggen et al. [48,49,50]
Rat hepatocytes	Fluorescence	R-123 130 nM	N. S.	Not tested	Not tested	Cavazzoni et al. [55]
Mouse lymphocytes	Fluorescence	DioC_3_(6) 20 nM	30%	Cyclosporin	Yes	Rottenberg and Wu [56]
Mouse T-cells	Fluorescence	DioC_3_(6) 20 nM	>50%	Cyclosporin	Yes	Mather and Rottenberg [57]
PC12 cells	Fluorescence	CMTMR (Q)	>50%	Cyclosporin	Yes	Sugrue et al. [58]
Rat cardio myocytes	Fluorescence	JC-1 4 μM (Q)	>50%	liraglutide	Not tested	Durak and Turan [59]
Drosophila gut stem cells	Fluorescence	TMRE 20 nM	30%	Not tested	Not tested	Morris et al. [60]
Mouse hematopoietic stem cells	Fluorescence	TMRM 100 nM(Q)	85% cells with low potential	MitoQ	Not tested	Mansell et al. [61]
Porcine oocytes	Fluorescence	JC-1	Reduced red/green ratio from 3 to 2	Quercetin	Not tested	Jiao et al. [64]
Rat cardio monocytes	Fluorescence	JC-1	Reduced red/green ratio from 0.32 to 0.28	SS-31	Yes	Zhang et al. [65]
*C. elegans* mitochondria	Fluorescence	DASPMI 6 μM (Q)	Reduced ± FCCP ratio from 1.75 to 1.25	Not tested	Not tested	Brys et al. [66]
*C. elegans* pharyngal bulb	Fluorescence	TMRE 1 μM (Q)	Reduced ~25% in gas-1 mutant	Not tested	Not tested	Dingley et al. [67]
*C. elegans* pharyngal bulb	Fluorescence	TMRE 100 nM (Q)	~0% reduction in gas-1 mutant	Not tested	Not tested	Kwan et al. [68]
*C. elegans* mitochondria	Fluorescence	R-123 (Q)	~50% reduction by Paraquat	Not tested	Not tested	Dilberger et al. [69]
*C. elegans* pharyngal bulb	Fluorescence	TMRE 100 nM (Q)	50–75% reduction	Optogenetic generation of ∆Ψm	Not tested	Berry et al. [25]
*C. elegans* pharyngal bulb	Fluorescence	TMRE 1 μM (Q)	~50% reduction	Dietary restriction	Not tested	Berry et al. [26]

## 3. Mitochondrial Membrane Potential and Aging in *C. elegans*

In recent years the worm *C. elegans* has become an important system for studies of animal aging. The very short lifespan and the small number of cells of this simple organism have enabled the relatively fast identification of many genes that either increase or decrease the worm lifespan, and the identification of many pharmacological agents that affect lifespan positively or negatively.

Brys et al. [66] studied the effect of the *C. elegans* daf-2 mutation on mitochondrial bioenergetics. They found that the daf-2 mutation, that encodes the insulin/insulin growth factor-1-like receptor and was shown to extend *C. elegans* lifespan, retarded the deterioration in mitochondrial bioenergetics in aging worms. In particular, they showed that, while ∆Ψm declined with age in wild-type worms, the daf-2 mutation increased ∆Ψm and largely retarded the decline of ∆Ψm with age. Brys et al. measured ∆Ψm in isolated mitochondria with the fluorescence dye DASPMI. They used a high concentration (~6 μM) of DASPMI, most likely in the quenching range. At day 1, the fluorescence ratio-control/+ FCCP (a measure of the magnitude of ∆Ψm) was 1.75. However, by day 6, this ratio was dramatically reduced to 1.20. In contrast, in the daf-2 mutants, the ratio at day 6 was 1.60, not significantly different from the 1.75 ratio that was observed in wt at day 1. Because Brys et al. [66] used a very high concentration of DASPMI, it is unlikely that the fluorescence ratio was proportional to the magnitude of ∆Ψm. Most likely, the lower ratio at old age indicates that there was an age-dependent increase in the number of depolarized mitochondria and that this effect of aging was inhibited in the daf-2 mutant. It has been shown previously that lifespan-extension of the daf-2 mutant is associated with a reduction in the level of VDAC1, which could lead to inhibition of mPTP [70], and it is, therefore, possible that the effect of the daf-2 mutation on ∆Ψm reflected the inhibition of mPTP in this mutant.

Dingley et al. [67] determined ∆Ψm in whole worms by including 1 μM TMRE in the growth medium. Before fluorescence measurement, the worms were washed to clear the dye from the intestine and the TMRE fluorescence was measured from the terminal paryngal bulb where the density of mitochondria is high. They compared TMRE fluorescence in the wild type (N2) with that of the gas-1 mutant. The gas-1 mutant serves as a model for accelerated aging since it carries a mutation in a complex I peptide that increases mROS production. The gas-1 mutant exhibited increased oxidative stress and shorter lifespan relative to N2. TMRE fluorescence was decreased by 63% in the gas-1 mutant, suggesting much lower ∆Ψm. However, Dingley et al. [67] also separately measured mitochondrial density with mitotracker green PM and showed that the density of the mitochondria was reduced by 48% in the gas-1 mutant. Thus, the reduction in TMRE fluorescence resulted largely from the reduction in mitochondrial density. If we correct for the reduction in mitochondrial density, the fluorescence from the mitochondria in the gas-1 mutation was ~15% lower than that of N2. Dingley et al. [67] incubated the worms for 24 h with 1 μM TMRE, which, even after washing the worms, was expected to be well into the quenching range. Experiments with isolated *C. elegans* mitochondria have shown that, even with 0.3 μM TMRE in the medium, the dye in the mitochondria was largely quenched [71], whereas it was necessary to use only 20 nM of TMRE in the mitochondrial suspension to avoid aggregation and quenching [72]. Therefore, the 25% reduction in fluorescence in the Dingley et al. experiments suggests that ~15% of the mitochondria in the gas-1 mutant were depolarized. It was previously shown by flow cytometry of isolated *C. elegans* mitochondria that under any condition a fraction of the mitochondria is depolarized [43]. It is, therefore, quite likely that enhancement of mPTP by the excess mROS generated by the gas-1 mutant increased the fraction of depolarized mitochondria. This may also explain the reduction in the mitochondrial density since depolarized mitochondria are expected to be cleared by mitophagy [16], and cells with a high number of depolarized mitochondria may proceed to apoptosis [32]. More recently Kwon et al. [68] found that the reduction in TMRE fluorescence in the gas-1 mutant could be accounted for entirely by the reduction in mitochondrial density.

Lemire et al. [73] also studied ∆Ψm in *C. elegans* mutants that affected the *C. elegans* lifespan. They used the lipophilic cation diSC_3_(3) at high concentration (4 μM) to measure ∆Ψm in several strains that had extended *C. elegans* lifespan (i.e., daf-2, age-1, clk-1, isp-1 and eat-2). At high concentration diSC_3_(3), aggregates in the mitochondria and the fluorescence maximum shifted to the red. Lemire et al. determined the fluorescence maximum of diS-C_3_(3) in whole worms at the larval stage (L4) of the various life-extending strains and found that at the larval stage, ∆Ψm, as estimated from the fluorescence maximum shift, was lower in all the life-extending strains compared to that of the wild type (N2). These results appear to contradict the results of Brys et al. [66], but it should be emphasized that these measurements were undertaken with the whole larva and not with mitochondria isolated from the adult worms; there are significant differences between the properties of adult and larval mitochondria [43]. Moreover, disruption of mitochondrial function during development was shown to increase lifespan [74].

Cho et al. [75] studied the effect of flavonoids on neurodegeneration in aging *C. elegans*. They found that flavonoids mitigate neurodegeneration in aging *C. elegans* and that this effect was associated with an early reduction in ∆Ψm. They measured ∆Ψm with TMRE at 100 nM in whole worms at the larval stage (L4). The effect of flavonoids on ∆Ψm was comparable to the weak uncoupler DNP, suggesting that this uncoupling effect enhanced mitophagy, which protected the worms against neurodegeneration. Uncouplers are known activators of the mPTP [76], and mPTP activation enhances mitophagy [16]. Thus, activating mPTP at an early age could lead to activation of protection mechanisms, such as mitophagy or the mitochondria unfolded protein response, UPRmt (see below). Cho et al. [75] also reported that ∆Ψ_m_ was much lower in the adult worms than in the larval state regardless of the flavonoids but did not present the evidence.

Dilberger et al. [69] studied the effects of paraquat on *C. elegans* longevity, mROS production and ∆Ψm. Paraquat is known to induce excess mROS production, as in aging, and can, therefore, be considered a pharmacological model of aging in *C. elegans*. Indeed, Dilberger et al. [69] showed that paraquat, depending on the concentration, greatly reduced *C. elegans* lifespan. This was associated with greatly increased mROS production, mitochondrial fragmentation and greatly reduced ∆Ψm. Dilberger et al. [69] measured ∆Ψm in isolated mitochondria from the fluorescence quenching of R123; paraquat (5 mM) reduced ∆Ψm by ~50%. In addition, they showed that paraquat increased mitochondrial fragmentation. Paraquat is known to induce the activation of mPTP [77], which is known to increase mitochondrial fragmentation in *C. elegans* [70]; therefore, the reduction in ∆Ψm by paraquat and the resulting reduction in lifespan can be attributed to the activation of mPTP.

Since paraquat induces the mitochondrial unfolded protein response, UPRmt, in *C. elegans* [24,78], the results of Dilberger et al. [69] suggest that the activation of mPTP is sufficient to activate UPRmt. Berry et al. [24] also showed that reduction in ∆Ψm by the uncoupler FCCP was sufficient to activate UPRmt in C. elegans. However, FCCP is known to activate mPTP [75], and Angeli et al. [79] showed that genetic or pharmacological inhibition of mPTP inhibited UPRmt. Moreover, it was recently shown that two signals from the mitochondria to the cytosol induced UPRmt, excess mROS and the accumulation of mitochondrial protein precursors [80]. Excess mROS was released by the activation of mPTP, and the depolarization of mitochondria resulting from the activation of mPTP inhibited the uptake of mitochondrial protein precursors, providing further support for the conclusion that activation of UPRmt depends on the activation of mPTP.

Interestingly, in their study, Berry et al. [24] measured ∆Ψm from TMRE fluorescence from the whole body of the worms after incubating the worms for 24 h with 100 nM TMRE. It appears, however, that at this high TMRE concentration, the dye in the mitochondrial matrix was largely aggregated and largely quenched. This can be deduced from the parallel titrations of the effect of FCCP on TMRE fluorescence and the activation of UPRmt. In the very wide range of FCCP concentrations from 10 pM to 10 μM, UPRmt was activated only at a narrow range around 10 nM, presumably resulting in a small reduction in ∆Ψm. However, the titration of TMRE fluorescence over the entire range of FCCP concentration did not show the expected linear titration curve. Already, at the very low concentration of 10 pM, there was a ~30% reduction in TMRE fluorescence but increasing concentrations even up to 3 uM did not reduce the fluorescence further. Only at 25 μM, presumably when the mitochondria became completely depolarized, was the fluorescence significantly reduced (~80%). Thus, the TMRE fluorescence values at 10 pM FCCP and 1 nM FCCP, which were insufficient to activate UPRmt, and the TMRE fluorescence value at 10 nM FCCP, that was sufficient to activate UPRmt, were all the same, demonstrating that, at this concentration of TMRE, the fluorescence magnitude over nearly the entire range of ∆Ψm values was not sensitive to the magnitude of ∆Ψm, and only when the mitochondria were depolarized was the TMRE fluorescence reduced as expected.

Berry et al. [71] targeted a fungal light-driven proton pump to the mitochondria of *C. elegans* to selectively increase the mitochondrial protonmotive force. They called this optogenetic tool mtON. They used TMRE at a high quenching concentration (300 nM) to measure ∆Ψm in isolated mitochondria from the fluorescence quenching of the suspending medium. They showed that in isolated non-respiring mitochondria, applying light and all trans retinal (ATR), the catalytic center of the light-driven proton pump, resulted in the generation of ∆Ψm that was collapsed by the uncoupler CCCP. Using the pH indicator BCECF, they also showed that mtON generated a pH gradient as expected. The ∆Ψm that was generated by the substrate succinate (in the presence of rotenone, “state 2”) was not significantly different from that generated by mtON at maximal illumination. However, applying both succinate and mtON did not increase ∆Ψm above the value that was generated by either succinate or mtON alone, suggesting that the magnitude of ∆Ψm in state 2 was limited by the membrane proton permeability. Berry et al. [71] also showed that mtON alone (without succinate) was able to generate ATP, resembling ATP generated by succinate oxidation. In “state 3” (i.e., succinate + ADP), mtON inhibited respiration by 50%, presumably because mtON was able to increase ∆Ψm in state 3 (however, Berry et al. [71] did not measure ∆Ψm in “state 3”). Electron transport inhibitors (i.e., rotenone, antimycin A and azide) killed most *C. elegans* worms within hours, presumably because they collapse ∆Ψm, and mtON was able to significantly increase worm survival in the presence of electron transport inhibitors. Finally, Berry et al. [71] showed that turning on mtON in the gas-1 mutant significantly increased motility that was otherwise greatly reduced in the mutant compared to wt. Since the reported reduction in ∆Ψm in the gas-1 mutant [68] undoubtedly resulted from the enhancement of the activation of mPTP by the excess mROS produced by the gas-1 mutation (see above), the restoration of motility to the gas-1 mutant by mtON could be attributed to inhibition of the voltage-gated mPTP by the ∆Ψm that was generated by mtON.

mtON should be an effective inhibitor of mPTP not only because high ∆Ψm decreases the probability of opening of the voltage-gated mPTP, but also, and perhaps more importantly, because it enables the channel to close quickly after releasing the excess calcium and mROS that activate the channel opening. Normally, electron transport proton pumps cannot easily restore ∆Ψm to close the channel because respiratory substrates are also released by the channel and electron transport is inhibited. However, the mtON-generated ∆Ψm is not dependent on electron transport and can, therefore, close the channel quickly after a relatively short opening.

Very recently, Berry et al. [25] showed that the optogenetic generation of ∆Ψm, (mtON) in *C. elegans* during the entire adult lifespan increased *C. elegans* lifespan. They also showed that both 4-day-old and 10-day-old worms increased thrashing in liquid (but not in solids) when mtON was activated, demonstrating that muscle activity, that normally deteriorates in old worms, can be partially rescued by ∆Ψm generated by mtON. In this study, they estimated ∆Ψm not in isolated mitochondria, but from the whole worm incubated with TMRE (100 nM). The TMRE fluorescence was collected from the head or the pharyngal bulbs that have a high concentration of mitochondria. They showed that, compared to TMRE fluorescence from the worms at day 1, TMRE fluorescence was greatly reduced with aging with a ~75% reduction in day 4 and a ~50% reduction in day 10. Berry et al. measured mitochondrial density at day 4 but not at day 1, so the reduction in TMRE fluorescence was most likely due, partially at least, to decreased mitochondrial density and partially due to decreased ∆Ψm (cf. [68]). In a separate experiment, with 4-day-old worms, Berry et al. [25] showed that turning on mtON greatly increased median TMRE fluorescence. In this experiment, they separately measured mitochondrial density with mitotracker and normalized the TMRE fluorescence to the mitochondrial density.

As discussed above, at 100 nM TMRE, the probe was aggregated and quenched in the mitochondrial matrix, so it was not sensitive to small changes in ∆Ψm, and the large reduction in fluorescence in aging worms observed was most likely due to an increased fraction of depolarized mitochondria and/or mitophagy-cleared depolarized mitochondria. If the average ∆Ψm in the mitochondria of aged worms was 50–75% lower than that of young worms, as claimed by Berry et al. [25], it would not be expected that an uncoupler like FCCP would stimulate respiration in old worms since the enhancement of respiration by an uncoupler results from the collapse of the protonmotive force. However, the fact that Berry et al. [25] observed no difference in the effect of FCCP on respiration between young and old worms suggests that there was no difference in the ∆Ψm of **respiring** mitochondria in old and young cells, and the lower TMRE fluorescence resulted from lower mitochondria density and/or a larger fraction of depolarized mitochondria that did not respire. The explanation that the low ∆Ψm that was observed in old worms resulted from enhanced activation of mPTP, and that mtON increased the lifespan and healthspan by inhibiting mPTP, is compatible with all the observations reported by Berry et al. [25].

In a more recent report, Berry et al. [26] investigated the relationship between dietary restriction (DR) that extended lifespan in *C. elegans* and ∆Ψm. Here, they showed again that aging from day 1 to day 4 was associated with a ~50% reduction in TMRE fluorescence under normal dietary conditions, while, when on DR, the reduction in TMRE fluorescence was much smaller, ~20%. In this study, they also measured TMRE fluorescence from the pharyngal bulb of whole worms, but in this case, they incubated the worms with 1 μM TMRE compared to 100 nM in their previous study. They did not explain this change of protocol, but it is clear that, under these new conditions, TMRE fluorescence from the mitochondrial matrix is in full quenching mode and the fluorescence cannot be proportional to the magnitude of ∆Ψm (see above). Therefore, in this study as well, the reduction in TMRE fluorescence in aged cells could indicate either a reduction in the density of mitochondria or an increase in the number of depolarized mitochondria, most likely both. Berry et al. [26] also found that several genetic and pharmacological manipulations modulated the effect of DR on longevity and TMRE fluorescence. The uncoupler FCCP inhibited the effect of DR on both longevity and TMRE fluorescence as expected. The mutant eat −2, a genetic model of DR, increased TMRE fluorescence at day 4 and extended lifespan. This effect was also inhibited by FCCP. a deletion of the uncoupling protein, unc-p, a deletion of the adenine nucleotide translocases ant-1,2, and the deletion of the IF1 protein (an ATP synthase inhibitor) mal-2. All raised the TMRE fluorescence at day 1, but only ant-1,2 and mal-2 inhibited the reduction in TMRE fluorescence at day 4 and extended lifespan. While the authors sought to explain these effects as resulting from modulations of the bioenergetic functions of mitochondria, all these effects can be attributed directly to the activation or inhibition of mPTP. It was shown that life extension by the eat-2 mutant depended on the inhibition of mPTP [67], and that FCCP was an activator of mPTP [76]. ANT 1 and ANT 2 are components of mPTP [18] and their deletion inhibited mPTP [70]. The protein IF1 interacts with ATP synthase, another component of mPTP [18], and was shown to activate mPTP [81]; therefore, the deletion of mal-2 is expected to inhibit mPTP. Thus, all the genetic and pharmacological effects on ∆Ψm that are described by Berry et al. [26] can be attributed to activation or inhibition of mPTP. Finally, cyclosporin A, an inhibitor of mPTP was shown to extend lifespan in *C. elegans* [82], and it was also shown that mPTP was inhibited in DR in mammals [83,84,85].

## 4. Conclusions

Mitochondrial dysfunction is a well-established “hallmark of aging”, which strongly interacts with most other “hallmarks of aging”. However, there are various manifestations of mitochondrial dysfunction in aging, which raises two questions: what are the causes of the mitochondrial dysfunctions that are associated with aging, and what are the consequences for health and longevity for each of the aging-associated mitochondrial dysfunctions? As discussed in detail in this review, one of the most frequently reported mitochondrial dysfunctions in aging is the reduction in ∆Ψm that is observed in many types of aging cells, but the cause of this effect is still not established. It was suggested previously that this may be the result of oxidative damage to mitochondrial phospholipids, such as cardiolipin [48]. Such damage could increase proton permeability and, thus, reduce ∆Ψm [15]. However, there is no evidence that the mitochondria of old cells are uncoupled. Alternatively, it was suggested that the lower ∆Ψm in aging is a result of an aging-specific metabolic state that impairs bioenergetic functions, and that this leads to disease and a shortened lifespan [23,24,25,26,27]. However, it was not specified what exactly this metabolic state is and how aging leads to this state.

Here we offer a new interpretation: the lower ∆Ψm that is observed in aging cells results from the enhanced activation of the mPTP, which increases the fraction of depolarized mitochondria. This interpretation is compatible with all the evidence reviewed here and is strongly supported by many of the reviewed studies. Several studies demonstrated that inhibition of mPTP restored ∆Ψm in aging cells (Table 1). Most of the studies that reported aging-associated reduction in ∆Ψm were conducted with whole cells, tissues, or even intact animals (e.g., worms). However, most of these studies employed high concentrations of fluorescent ∆Ψm indicators in which the fluorescence was in the quenching mode and the fluorescence was not proportional to the magnitude of ∆Ψm. Therefore, the fluorescence was only proportional to the number of mitochondria with significant ∆Ψm. Any reduction in probe fluorescence in aging cells under these assay conditions simply indicates loss of mitochondria with ∆Ψm, either due to depolarization or elimination of depolarized mitochondria by mitophagy, both of which could be the result of mPTP activation. Only a few studies measured mitochondria density with an appropriate probe, and these suggest that aging is associated with both a reduction in mitochondrial density and an increased fraction of depolarized mitochondria. Many of the studies that reported lower ∆Ψm in aged cells also reported enhanced activation of mPTP (Table 1), which supports this interpretation of the results. Of all the studies of the reduction in ∆Ψm in whole aging cells, only the study of Morris et al. [60] that reported a 20% lower average ∆Ψm in aging intestinal stem cells from *Drosophila*, used a valid protocol for ∆Ψm measurement in whole cells, with a low non-quenching TMRE concentration (20 nM), and where the fluorescence was collected directly from within the labeled mitochondria. However, Morris et al. showed that the old intestinal stem cells in *Drosophila* were in a very distinct metabolic state with a largely inhibited mitochondrial calcium transporter, MCU, which could explain the lower ∆Ψm. This state was not reported in any other aging cells study and does not appear to represent a general feature of mitochondrial dysfunction in aging cells.

Several experiments with isolated mitochondria from tissues of aged animals showed a small reduction (~10%) in the average ∆Ψm. While the protocols of these experiments are more reliable, these results are also compatible with the suggestion that in aging cells there is a larger fraction of depolarized mitochondria. Whether this is the case or not can only be decided by measuring ∆Ψm of individual mitochondria (cf. [43]). If there was really a significant reduction in the average ∆Ψm in aging cells, it would be expected that there would be a reduction in mROS production and increased uncoupling, but this is not observed in aging cells—it is widely reported that mROS production is increased in aged cells and that the coupling is not impaired.

The conclusion that the reduction in ∆Ψm in aging cells results from mitochondrial depolarization by the enhanced activation of mPTP in aging cells provides a straightforward explanation of all the reported effects of ∆Ψm on aging. Enhanced activation of mPTP leads to excess release of mROS and destruction of NAD^+^ and this leads to many of the reported effects of aging (Figure 1), to age-dependent degenerative diseases, and, eventually, to cell death by necrosis or apoptosis. On the other hand, limited activation of mPTP, particularly at an early age, initiates protective mechanisms, such as UPRmt, mitophagy, and mitochondria biogenesis. Inhibition of mPTP activation by dietary restriction, drugs or artificially produced ∆Ψm, retards the process of aging, inhibits degenerative diseases, and increases longevity.

## Data Availability

No new data created in this review.

## References

[1] López-Otín C., Blasco M.A., Partridge L., Serrano M., Kroemer G. (2013). The hallmarks of aging. Cell.

[2] López-Otín C., Blasco M.A., Partridge L., Serrano M., Kroemer G. (2023). Hallmarks of aging: An expanding universe. Cell.

[3] Gómez J., Mota-Martorell N., Jové M., Pamplona R., Barja G. (2023). Mitochondrial ROS production, oxidative stress and aging within and between species: Evidences and recent advances on this aging effector. Exp. Gerontol..

[4] Maldonado E., Morales-Pison S., Urbina F., Solari A. (2023). Aging Hallmarks and the Role of Oxidative Stress. Antioxidants.

[5] Shadfar S., Parakh S., Jamali M.S., Atkin J.D. (2023). Redox dysregulation as a driver for DNA damage and its relationship to neurodegenerative diseases. Transl. Neurodegener..

[6] Moustakli E., Zikopoulos A., Sakaloglou P., Bouba I., Sofikitis N., Georgiou I. (2023). Functional association between telomeres, oxidation and mitochondria. Front. Reprod. Health.

[7] Trigo D., Nadais A., Carvalho A., Morgado B., Santos F., Nóbrega-Pereira S., e Silva O.A.B.d.C. (2023). Mitochondria dysfunction and impaired response to oxidative stress promotes proteostasis disruption in aged human cells. Mitochondrion.

[8] Luo H., Chiang H.H., Louw M., Susanto A., Chen D. (2017). Nutrient Sensing and the Oxidative Stress Response. Trends Endocrinol. Metab..

[9] Fujita Y., Iketani M., Ito M., Ohsawa I. (2022). Temporal changes in mitochondrial function and reactive oxygen species generation during the development of replicative senescence in human fibroblasts. Exp. Gerontol..

[10] Andrade B., Jara-Gutiérrez C., Paz-Araos M., Vázquez M.C., Díaz P., Murgas P. (2022). The Relationship between Reactive Oxygen Species and the cGAS/STING Signaling Pathway in the Inflammaging Process. Int. J. Mol. Sci..

[11] Mantel C., Messina-Graham S., Moh A., Cooper S., Hangoc G., Fu X.Y., Broxmeyer H.E. (2012). Mouse hematopoietic cell-targeted STAT3 deletion: Stem/progenitor cell defects, mitochondrial dysfunction, ROS overproduction, and a rapid aging-like phenotype. Blood.

[12] Pan C., Zhou F., Zhang L. (2023). The loss of epigenetic information: Not only consequences but a cause of mammalian aging. Signal Transduct. Target. Ther..

[13] Harman D. (1972). The biologic clock: The mitochondria?. J. Am. Geriatr. Soc..

[14] Barja G. (2014). The mitochondrial free radical theory of aging. Prog. Mol. Biol. Transl. Sci..

[15] Rottenberg H., Hoek J.B. (2017). The path from mitochondrial ROS to aging runs through the mitochondrial permeability transition pore. Aging Cell.

[16] Rottenberg H., Hoek J.B. (2021). The Mitochondrial Permeability Transition: Nexus of Aging, Disease and Longevity. Cells.

[17] Bernardi P., Krauskopf A., Basso E., Petronilli V., Blachly-Dyson E., Di Lisa F., Forte M.A. (2006). The mitochondrial permeability transition from in vitro artifact to disease target. FEBS J..

[18] Carraro M., Carrer A., Urbani A., Bernardi P. (2020). Molecular nature and regulation of the mitochondrial permeability transition pore(s), drug target(s) in cardioprotection. J. Mol. Cell Cardiol..

[19] Di Lisa F., Menabò R., Canton M., Barile M., Bernardi P. (2001). Opening of the mitochondrial permeability transition pore causes depletion of mitochondrial and cytosolic NAD+ and is a causative event in the death of myocytes in postischemic reperfusion of the heart. J. Biol. Chem..

[20] Covarrubias A.J., Perrone R., Grozio A., Verdin E. (2021). NAD^+^ metabolism and its roles in cellular processes during ageing. Nat. Rev. Mol. Cell Biol..

[21] Sugrue M.M., Tatton W.G. (2001). Mitochondrial membrane potential in aging cells. Biol. Signals Recept..

[22] Nicholls D.G. (2004). Mitochondrial membrane potential and aging. Aging Cell.

[23] Berry B.J., Kaeberlein M. (2021). An energetics perspective on geroscience: Mitochondrial protonmotive force and aging. Geroscience.

[24] Berry B.J., Nieves T.O., Wojtovich A.P. (2021). Decreased Mitochondrial Membrane Potential Activates the Mitochondrial Unfolded Protein Response. microPublication Biol..

[25] Berry B.J., Vodičková A., Müller-Eigner A., Meng C., Ludwig C., Kaeberlein M., Peleg S., Wojtovich A.P. (2023). Optogenetic rejuvenation of mitochondrial membrane potential extends *C. elegans* lifespan. Nat. Aging.

[26] Berry B.J., Mjelde E., Carreno F., Gilham K., Hanson E.J., Na E., Kaeberlein M. (2023). Preservation of mitochondrial membrane potential is necessary for lifespan extension from dietary restriction. GeroScience.

[27] Berry B.J., Pharaoh G.A., Marcinek D.J. (2023). From mitochondria to cells to humans: Targeting bioenergetics in aging and disease. Int. J. Biochem. Cell Biol..

[28] Zorova L.D., Popkov V.A., Plotnikov E.Y., Silachev D.N., Pevzner I.B., Jankauskas S.S., Babenko V.A., Zorov S.D., Balakireva A.V., Juhaszova M. (2018). Mitochondrial membrane potential. Anal. Biochem..

[29] Divakaruni A.S., Brand M.D. (2011). The regulation and physiology of mitochondrial proton leak. Physiology.

[30] Chenna S., Koopman W.J.H., Prehn J.H.M., Connolly N.M.C. (2022). Mechanisms and mathematical modeling of ROS production by the mitochondrial electron transport chain. Am. J. Physiol. Cell Physiol..

[31] Vasan K., Clutter M., Fernandez Dunne S., George M.D., Luan C.H., Chandel N.S., Martínez-Reyes I. (2022). Genes Involved in Maintaining Mitochondrial Membrane Potential upon Electron Transport Chain Disruption. Front. Cell Dev. Biol..

[32] Bonora M., Giorgi C., Pinton P. (2022). Molecular mechanisms and consequences of mitochondrial permeability transition. Nat. Rev. Mol. Cell Biol..

[33] Crompton M. (2004). Mitochondria and aging: A role for the permeability transition?. Aging Cell.

[34] Di Lisa F., Bernardi P. (2005). Mitochondrial function and myocardial aging. A critical analysis of the role of permeability transition. Cardiovasc. Res..

[35] Toman J., Fiskum G. (2011). Influence of aging on membrane permeability transition in brain mitochondria. J. Bioenerg. Biomembr..

[36] Paradies G., Paradies V., Ruggiero F.M., Petrosillo G. (2013). Changes in the mitochondrial permeability transition pore in aging and age-associated diseases. Mech. Ageing Dev..

[37] Pellegrino-Coppola D. (2020). Regulation of the mitochondrial permeability transition pore and its effects on aging. Microb. Cell.

[38] Rottenberg H. (1979). The measurement of membrane potential and deltapH in cells, organelles, and vesicles. Methods Enzymol..

[39] Rottenberg H., Wu S. (1998). Quantitative assay by flow cytometry of the mitochondrial membrane potential in intact cells. Biochim. Biophys. Acta.

[40] Nicholls D.G., Ward M.W. (2000). Mitochondrial membrane potential and neuronal glutamate excitotoxicity: Mortality and millivolts. Trends Neurosci..

[41] Lerner C.A., Gerencser A.A. (2022). Unbiased Millivolts Assay of Mitochondrial Membrane Potential in Intact Cells. Methods Mol. Biol..

[42] Chazotte B. (2011). Labeling mitochondria with JC-1. Cold Spring Harb. Protoc..

[43] Daniele J.R., Heydari K., Arriaga E.A., Dillin A. (2016). Identification and Characterization of Mitochondrial Subtypes in *Caenorhabditis elegans* via Analysis of Individual Mitochondria by Flow Cytometry. Anal. Chem..

[44] Kokoszka J.E., Coskun P., Esposito L.A., Wallace D.C. (2001). Increased mitochondrial oxidative stress in the Sod2 (+/−) mouse results in the age-related decline of mitochondrial function culminating in increased apoptosis. Proc. Natl. Acad. Sci. USA.

[45] Mather M., Rottenberg H. (2000). Aging enhances the activation of the permeability transition pore in mitochondria. Biochem. Biophys. Res. Commun..

[46] LaFrance R., Brustovetsky N., Sherburne C., Delong D., Dubinsky J.M. (2005). Age-related changes in regional brain mitochondria from Fischer 344 rats. Aging Cell.

[47] Gainutdinov T., Gizatullina Z., Debska-Vielhaber G., Vielhaber S., Feldmann R.E., Orynbayeva Z., Gellerich F.N. (2023). Age-associated alterations of brain mitochondria energetics. Biochem. Biophys. Res. Commun..

[48] Hagen T.M., Yowe D.L., Bartholomew J.C., Wehr C.M., Do K.L., Park J.Y., Ames B.N. (1997). Mitochondrial decay in hepatocytes from old rats: Membrane potential declines, heterogeneity and oxidants increase. Proc. Natl. Acad. Sci. USA.

[49] Hagen T.M., Ingersoll R.T., Lykkesfeldt J., Liu J., Wehr C.M., Vinarsky V., Bartholomew J.C., Ames A.B. (1999). (R)-alpha-lipoic acid-supplemented old rats have improved mitochondrial function, decreased oxidative damage, and increased metabolic rate. FASEB J..

[50] Hagen T.M., Liu J., Lykkesfeldt J., Wehr C.M., Ingersoll R.T., Vinarsky V., Bartholomew J.C., Ames B.N. (2002). Feeding acetyl-L-carnitine and lipoic acid to old rats significantly improves metabolic function while decreasing oxidative stress. Proc. Natl. Acad. Sci. USA.

[51] Miwa S., Kashyap S., Chini E., von Zglinicki T. (2022). Mitochondrial dysfunction in cell senescence and aging. J. Clin. Investig..

[52] Korshunov S.S., Skulachev V.P., Starkov A.A. (1997). High protonic potential actuates a mechanism of production of reactive oxygen species in mitochondria. FEBS Lett..

[53] Brand M.D., Buckingham J.A., Esteves T.C., Green K., Lambert A.J., Miwa S., Murphy M.P., Pakay J.L., Talbot D.A., Echtay K.S. (2004). Mitochondrial superoxide and aging: Uncoupling-protein activity and superoxide production. Biochem. Soc. Symp..

[54] Rottenberg H., Covian R., Trumpower B.L. (2009). Membrane potential greatly enhances superoxide generation by the cytochrome bc1 complex reconstituted into phospholipid vesicles. J. Biol. Chem..

[55] Cavazzoni M., Barogi S., Baracca A., Parenti Castelli G., Lenaz G. (1999). The effect of aging and an oxidative stress on peroxide levels and the mitochondrial membrane potential in isolated rat hepatocytes. FEBS Lett..

[56] Rottenberg H., Wu S. (1997). Mitochondrial dysfunction in lymphocytes from old mice: Enhanced activation of the permeability transition. Biochem. Biophys. Res. Commun..

[57] Mather M.W., Rottenberg H. (2002). The inhibition of calcium signaling in T lymphocytes from old mice results from enhanced activation of the mitochondrial permeability transition pore. Mech. Ageing Dev..

[58] Sugrue M.M., Wang Y., Rideout H.J., Chalmers-Redman R.M., Tatton W.G. (1999). Reduced mitochondrial membrane potential and altered responsiveness of a mitochondrial membrane megachannel in p53-induced senescence. Biochem. Biophys. Res. Commun..

[59] Durak A., Turan B. (2023). Liraglutide provides cardioprotection through the recovery of mitochondrial dysfunction and oxidative stress in aging hearts. J. Physiol. Biochem..

[60] Morris O., Deng H., Tam C., Jasper H. (2020). Warburg-like Metabolic Reprogramming in Aging Intestinal Stem Cells Contributes to Tissue Hyperplasia. Cell Rep..

[61] Mansell E., Sigurdsson V., Deltcheva E., Brown J., James C., Miharada K., Soneji S., Larsson J., Enver T. (2021). Mitochondrial Potentiation Ameliorates Age-Related Heterogeneity in Hematopoietic Stem Cell Function. Cell Stem Cell.

[62] Chen J., Zhang M., Zou H., Aniagu S., Jiang Y., Chen T. (2023). PM2.5 induces mitochondrial dysfunction via AHR-mediated cyp1a1 overexpression during zebrafish heart development. Toxicology.

[63] Daniel O.O., Adeoye A.O., Ojowu J., Olorunsogo O.O. (2018). Inhibition of liver mitochondrial membrane permeability transition pore opening by quercetin and vitamin E in streptozotocin-induced diabetic rats. Biochem. Biophys. Res. Commun..

[64] Jiao Y., Wang Y., Jiang T., Wen K., Cong P., Chen Y., He Z. (2022). Quercetin protects porcine oocytes from in vitro aging by reducing oxidative stress and maintaining the mitochondrial functions. Front. Cell Dev. Biol..

[65] Zhang H., Alder N.N., Wang W., Szeto H., Marcinek D.J., Rabinovitch P.S. (2020). Reduction of elevated proton leak rejuvenates mitochondria in the aged cardiomyocyte. eLife.

[66] Brys K., Castelein N., Matthijssens F., Vanfleteren J.R., Braeckman B.P. (2010). Disruption of insulin signalling preserves bioenergetic competence of mitochondria in ageing *Caenorhabditis elegans*. BMC Biol..

[67] Dingley S., Polyak E., Lightfoot R., Ostrovsky J., Rao M., Greco T., Ischiropoulos H., Falk M.J. (2010). Mitochondrial respiratory chain dysfunction variably increases oxidant stress in *Caenorhabditis elegans*. Mitochondrion.

[68] Kwon Y.J., Guha S., Tuluc F., Falk M.J. (2018). High-throughput BioSorter quantification of relative mitochondrial content and membrane potential in living *Caenorhabditis elegans*. Mitochondrion.

[69] Dilberger B., Baumanns S., Schmitt F., Schmiedl T., Hardt M., Wenzel U., Eckert G.P. (2019). Mitochondrial Oxidative Stress Impairs Energy Metabolism and Reduces Stress Resistance and Longevity of *C. elegans*. Oxidative Med. Cell. Longev..

[70] Zhou B., Kreuzer J., Kumsta C., Wu L., Kamer K.J., Cedillo L., Zhang Y., Li S., Kacergis M.C., Webster C.M. (2019). Mitochondrial Permeability Uncouples Elevated Autophagy and Lifespan Extension. Cell.

[71] Berry B.J., Trewin A.J., Milliken A.S., Baldzizhar A., Amitrano A.M., Lim Y., Kim M., Wojtovich A.P. (2020). Optogenetic control of mitochondrial protonmotive force to impact cellular stress resistance. EMBO Rep..

[72] Berry B.J., Baldzizhar A., Nieves T.O., Wojtovich A.P. (2020). Neuronal AMPK coordinates mitochondrial energy sensing and hypoxia resistance in *C. elegans*. FASEB J..

[73] Lemire B.D., Behrendt M., DeCorby A., Gásková D. (2009). *C. elegans* longevity pathways converge to decrease mitochondrial membrane potential. Mech. Ageing Dev..

[74] Munkácsy E., Rea S.L. (2014). The paradox of mitochondrial dysfunction and extended longevity. Exp. Gerontol..

[75] Cho I., Song H.O., Cho J.H. (2020). Flavonoids mitigate neurodegeneration in aged *Caenorhabditis elegans* by mitochondrial uncoupling. Food Sci. Nutr..

[76] Bernardi P. (1992). Modulation of the mitochondrial cyclosporin A-sensitive permeability transition pore by the proton electrochemical gradient. Evidence that the pore can be opened by membrane depolarization. J. Biol. Chem..

[77] Costantini P., Petronilli V., Colonna R., Bernardi P. (1995). On the effects of paraquat on isolated mitochondria. Evidence that paraquat causes opening of the cyclosporin A-sensitive permeability transition pore synergistically with nitric oxide. Toxicology.

[78] Kim S., Sieburth D. (2018). Sphingosine Kinase Activates the Mitochondrial Unfolded Protein Response and Is Targeted to Mitochondria by Stress. Cell Rep..

[79] Angeli S., Foulger A., Chamoli M., Peiris T.H., Gerencser A., Shahmirzadi A.A., Andersen J., Lithgow G. (2021). The mitochondrial permeability transition pore activates the mitochondrial unfolded protein response and promotes aging. eLife.

[80] Sutandy F.X.R., Gößner I., Tascher G., Münch C. (2023). A cytosolic surveillance mechanism activates the mitochondrial UPR. Nature.

[81] Guo L. (2022). Mitochondrial ATP synthase inhibitory factor 1 interacts with the p53-cyclophilin D complex and promotes opening of the permeability transition pore. J. Biol. Chem..

[82] Ye X., Linton J.M., Schork N.J., Buck L.B., Petrascheck M. (2014). A pharmacological network for lifespan extension in *Caenorhabditis elegans*. Aging Cell.

[83] Kristal B.S., Yu B.P. (1998). Dietary restriction augments protection against induction of the mitochondrial permeability transition. Free Radic. Biol. Med..

[84] Menezes-Filho S.L., Amigo I., Prado F.M., Ferreira N.C., Koike M.K., Pinto I.F.D., Miyamoto S., Montero E.F.S., Medeiros M.H.G., Kowaltowski A.J. (2017). Caloric restriction protects livers from ischemia/reperfusion damage by preventing Ca^2+^-induced mitochondrial permeability transition. Free Radic. Biol. Med..

[85] Amigo I., Menezes-Filho S.L., Luévano-Martínez L.A., Chausse B., Kowaltowski A.J. (2017). Caloric restriction increases brain mitochondrial calcium retention capacity and protects against excitotoxicity. Aging Cell.

